# Web-Based Passive Surveillance: Multifactorial Assessment of Sonali Chicken Diseases and Antimicrobial Prescription Pattern in Bangladesh

**DOI:** 10.3390/vetsci11120662

**Published:** 2024-12-17

**Authors:** Ibrahim Khalil, Md. Abu Sayeed, Mitun Sarkar, Md. Nurul Islam, Mozaffar G. Osmani, Meherjan Islam, Sharmin Chowdhury, Md. Abu Shoieb Mohsin, Md. Ahasanul Hoque

**Affiliations:** 1 Department of Livestock Services, Ministry of Fisheries and Livestock, Dhaka 1215, Bangladesh; ibrahim.khalil@dls.gov.bd (I.K.); mitun@bdvets.com (M.S.); muzaffar.osmani@dls.gov.bd (M.G.O.); 2 National Centre for Epidemiology and Population Health (NCEPH), College of Health and Medicine, The Australian National University, Canberra, ACT 2601, Australia; 3 The Eastern Mediterranean Public Health Network, Country Office, Niketon, Dhaka 1212, Bangladesh; mdnurul.islam@wisc.edu; 4 Faculty of Veterinary Medicine, Chattogram Veterinary and Animal Sciences University, Zakir Hossain Road, Khulshi, Chattogram 4225, Bangladesh; meherjanislam@gmail.com (M.I.); sharminchowdhury@cvasu.ac.bd (S.C.); shoieb.tox@gmail.com (M.A.S.M.); 5 One Health Institute, Chattogram Veterinary and Animal Sciences University, Zakir Hossain Road, Khulshi, Chattogram 4225, Bangladesh

**Keywords:** Sonali chickens, web-based passive surveillance, antimicrobial usage, climatic factor, Bangladesh

## Abstract

Despite the significant growth in Sonali chicken production across Bangladesh, inadequate disease surveillance and control measures along with indiscriminate antimicrobial use remain major challenges to the sector. In this study, we evaluated the disease burden and antimicrobial prescription patterns of Sonali chickens in Bangladesh using a web-based data recording system from 2020 to 2021 and analyzed 1690 cases. The diagnoses recorded in the system were presumptive, as they were based on clinico-epidemiological history, clinical signs, and gross necropsy findings noted by registered veterinarians. We conducted this study in Bogura, a district renowned for its high concentration of Sonali chicken farms. We estimated a higher prevalence of infection among grower chickens (69.0%) compared to starter chickens (31.0%). Small- to medium-sized flocks (63%) were more frequently infected than larger flocks (37.0%). Most disease cases occurred during the summer season (43.0%), followed by winter (27%), the rainy season (15%), and autumn (14%). Overall, climatic factors contributed to 51% of disease occurrence at temperatures below 25°C, 55% at high humidity (≥75%), and 57% during heavy rainfall (≥29 mm). The most prevalent disease was Newcastle disease (ND) (19.5%), followed by Marek’s disease (9.8%), coccidiosis (7.4%), necrotic enteritis (4.7%), infectious bursal disease (3.2%), and infectious laryngotracheitis (3.2%). The odds of ND were 1.4 (grower chickens vs. starter chickens), 11.4 (summer vs. winter), 4.1 (autumn vs. winter), 3.9 (rainy vs. winter), 3.5 (≥25 °C vs. <25 °C), and 2.6 (≥75% vs. <75%). Tylvalosin (38.0%) was the most frequently prescribed antibiotic, followed by fluoroquinolones (9.0%), aminoglycosides (8.0%), and colistin sulphate (4.0%). These findings suggest that a web-based disease record could be an important tool for a centralized poultry disease surveillance system in low- and middle-income countries like Bangladesh.

## Simple Summary

This study highlights the growing need for robust disease surveillance in the rapidly expanding Sonali chicken sector in Bangladesh, where improper disease management and overuse of antibiotics remain critical challenges. By analyzing 1690 cases through a web-based platform in Bogura district (small administrative region), we found significant links between seasonal and climatic factors and disease outbreaks, with Newcastle disease emerging as the most prevalent. This study underscores the importance of web-based data systems as vital tools for monitoring and controlling poultry diseases, offering a scalable solution for improving livestock health management in low- and middle-income countries like Bangladesh.

## 1. Introduction

In Bangladesh, the poultry subsector contributes to protein sourcing, direct and indirect employment generation, and economic input, adding 1.9% to the total country’s gross domestic product [1,2]. However, frequent poultry disease occurrences hinder the sector’s progress due to poorly structured, non-systematic, and untactful disease surveillance systems [3]. Poultry disease surveillance requires a poultry disease database, including loss estimation and assessment of required measures to prevent and control poultry diseases [4]. The Department of Livestock Services (DLS) has both manual and web-based data management systems in Bangladesh’s veterinary sector. The manual data management system primarily involves maintaining a record-keeping book in the government veterinary hospitals (GVH) at the sub-district level. The veterinarians at GVH record data on farmers’ complaints, clinical signs, post-mortem findings (particularly in poultry cases), and other findings in the record-keeping book, which also includes prescribed medicines and follow-up. In 2019, the DLS started to collaborate with the Food and Agriculture Organization of the United Nations (FAO) Emergency Centre for Transboundary Animal Diseases (ECTAD) for the development of the Bangladesh Animal Health Intelligence System (BAHIS), a web-based animal disease passive surveillance system. The initiators introduced this evidence-based data recording system with the aim of ensuring timely field reporting and analysis of received data, thereby assisting policymakers in controlling and preventing animal and poultry diseases [5]. However, several factors, such as a lack of online prescription features and a shortage of technical personnel in GVH, have affected the system, forcing it to remain in the testing phase [5].

Although private industries in Bangladesh’s poultry sector owned their individual web-based data management systems, the employees were used to maintain them for recording their company’s associated farm data [6]. Other private veterinarians cannot access these systems because their primary purpose is to serve company benefits, such as estimating feed efficacy and quality, and determine the prevalence of diseases in the day-old chickens supplied by the companies [6]. Therefore, bdvets.com has launched a private web-based passive surveillance platform with the aim of providing remote veterinary services to marginal farmers through an online prescription facility [7]. Unlike the BAHIS and private company-authorized websites, bdvets.com offers free access to all veterinarians (both government and private) and farmers. The system is also accessible through a dedicated mobile application, enhancing its practicality [7]. Manual data handling may have caused underreporting and delays in catching real-time disease outbreaks. Furthermore, existing study findings on poultry disease status are insufficient and dispersed, resulting in insufficient information about the disease’s epidemiology (disease patterns and distributions) in Bangladesh to implement suitable preventative and control measures [3,8]. This emphasizes the need for transitioning to a web-based system, such as bdvets.com, which can offer more timely and accurate disease surveillance and reporting. Therefore, for this study, we used the online data from the bdvets.com portal.

This study focused on data related to Sonali (Rhode Island Red cockerel × Fayoumi hen) chickens that were first introduced in the northern part of Bangladesh to empower women to reduce poverty [3,9]. Sonali meat consumption rose to 45% in 2019 due to its close resemblance and taste to the local indigenous chickens [3]. These chickens are reared for both meat and egg purposes, although the layer chickens are mostly farmed for hatching eggs to produce day-old chicks (DOCs) due to poor productivity of table eggs [10]. Small- and medium-scale farmers favor Sonali chickens above other strains because of their flexibility and productivity in scavenging and semi-scavenging rearing systems [11]. The longer rearing period and commercially intensive rearing system of Sonali chickens reduce their disease resistance capacity because of inbreeding depression [12]. In addition, geo-climatic calamities and inadequate farming management increase disease outbreaks, with high mortality and morbidity in commercial Sonali farms [13,14]. Many studies using manually managed data and cross-sectional surveys revealed a high prevalence of infectious diseases, such as protozoal, viral, and bacterial in Sonali chickens [3,15,16,17].

Sonali chickens are prone to viral disease, like ND, infectious bursal disease (IBD), fowl pox (FP), and low pathogenic avian influenza (LPAI) [16,18,19]. In Bangladesh, infectious diseases including ND kill up to 30% of birds annually [18]. ND seroprevalence is reported at 21.2% in domestic chickens [20] and 37.5% in commercial layers [21], with mortality rates of 15.81% in semi-scavenging layers [22] and 13.4% in commercial layers [22]. In Bangladesh, ND epidemiology is poorly understood despite its significant burden. This study examines the prevalence and risk factors associated with ND in commercial Sonali chicken farms.

Farmers commonly use antibiotics in the field to prevent and control poultry diseases [3]. In addition to registered veterinarians, feed dealers and farmers also participate in the administration of various antimicrobials [23] for both therapeutic and prophylactic purposes [24]. Data management and disease surveillance issues prevent a nationwide census of rising antimicrobial resistance (AMR) in the veterinary sector [25]. However, the majority of the data came from four veterinary sentinel laboratories: the Field Disease Investigation Laboratory (FDIL) in Feni and Joypurhat, the Bangladesh Livestock Research Institute (BLRI), the Poultry Research and Training Centre (PRTC) laboratory at Chattogram Veterinary and Animal Sciences University (CVASU), and the Central Disease Investigation Laboratory (CDIL) [26]. Despite these laboratory-based surveillance programs, an actual summary of antimicrobial prescribing patterns in Bangladesh remains lacking [25]. Therefore, in this study, we also investigated the antimicrobial prescription patterns through analysis of the web-based data.

## 2. Materials and Methods

### 2.1. Study Location

Bogura, a district comprising 12 Upazilas (sub-districts), often referred to as the gateway to North Bengal, is situated between 24°32′ and 25°07′ N latitude and 88°58′ and 89°45′ E longitude, covering an area of 2898.68 square kilometers [27,28,29]. In Bogura, livestock farming is one of the predominant occupations, followed by public service, general commerce, customer service, transportation, wage labor, and various other industries [27]. The average annual dry bulb temperature, maximum and minimum temperature, rainfall, and humidity in 2020 and 2021 were 25.8 °C, 37.8 °C, 7.7 °C, 1855 mm, and 76% [30]. The enlisted poultry farm distribution of Bogura is 480,494 deshi (local) chicken household farms, 1870 broiler farms, 1620 Sonali farms, 1123 layer farms, 584 duck farms, 8 breeder farms, and 67 hatcheries [31].

### 2.2. Study Design and Duration

We conducted a retrospective, cross-sectional study of Sonali chicken disease cases in Bogura district. This study focused on retrospective data recorded between January 2020 and December 2021, which included information on clinical signs, disease conditions, treatments administered, and the production challenges faced by farmers during this period.

### 2.3. Data Sources and Collection

#### 2.3.1. Sources and Collection of Case Data

We employed a non-probability purposive sampling approach in this study to assess disease prevalence and antimicrobial usage in a large population of diseased poultry, utilizing bdvets.com, a web-based data recording system. From the launch of the bdvets.com website in November 2019 until 2021, a total of 3795 cases were recorded. These comprised 2927 poultry cases, 726 large and small ruminant cases, and 142 pet cases. Of the poultry cases, 2195 involved Sonali chickens, and 732 involved broilers, with cases originating from districts such as Bogura, Joypurhat, Gaibandha, Dinajpur, Rangpur, Narsingdi, and Narayanganj. The high number of Sonali cases from Bogura (1690) led to the selection of Sonali cases as the study population. We utilized the diagnostic data provided by veterinarians on the bdvets.com platform. The website offered online and offline vet consultations through a prescheduled appointment system. Farmers could provide contact details and basic patient information (species, age, weight, symptoms) to book with a preferred or assigned veterinarian. An AI chatbot could also assist in scheduling by collecting this information. Farmers could communicate with the assigned veterinarian via the website or WhatsApp Messenger.

The diagnoses of diseases were primarily based on clinical signs and gross necropsy findings conducted by trained veterinarians [32,33,34]. Additionally, the assigned veterinarians integrated clinico-epidemiological history, prescription records, farmer interviews, and direct case observations into their diagnostic process. A standardized data collection form, hosted on the website, was completed for each case to ensure consistency and accuracy in data recording. This comprehensive diagnostic approach enabled a thorough analysis of the health status of Sonali chickens, the prevalence of various diseases, and patterns of antimicrobial usage within the study population.

#### 2.3.2. Sources and Collection of Meteorological Data

We collected climate data for this study from the Bangladesh Meteorological Department [30] for the period between January 2020 and December 2021. The data were sourced from the meteorological station in Bogura, which records daily measurements. We gathered daily climate data, including maximum, minimum, and mean temperatures, relative humidity (RH), and rainfall. The data were recorded on a day-by-day basis, providing a comprehensive climate profile for our study period.

### 2.4. Data Analysis

First, we exported all poultry case data from the bdvets.com website into Excel format using MS Excel 2010. We then sorted, coded, and cleaned the data specific to Sonali chicken cases to meet the requirements for analysis. The targeted Sonali chickens were reared in a litter-floored intensive farming system. To ensure that only relevant data were selected, we applied a detailed filtering process using specific markers to exclude non-Sonali cases. Once the data were cleaned and coded, we exported them to STATA/SE-13 (StataCorp, 4905 Lakeway Drive, College Station, TX 77845-4512, USA) for detailed epidemiological analysis. We conducted a descriptive analysis to calculate the frequency distribution of cases according to different demographic characteristics, including age, rearing stage, flock size, and climatic factors like temperature, humidity, and rainfall. We estimated the prevalence of different poultry diseases and associated conditions, with the frequency and percentage of different antibiotics prescribed for each case. To assess potential risk factors for the occurrence of ND, we first applied Fisher’s exact test, followed by univariate logistic regression. We considered factors such as age (starter, grower), flock size (small, medium, large), season (winter, summer, rainy, autumn), temperature (≥25 °C, <25 °C), humidity (≥75%, <75%), and rainfall (≥29 mm, <29 mm). We used 25 °C temperature, 75% humidity, and 29% rainfall as the cut-off due to the mean climate prevailing in the city of Bogura [35]. Due to heteroscedasticity (*p* ≤ 0.05 in the regression diagnosis tests), we excluded multivariate logistic regression, as the model did not meet the diagnostic criteria. The goodness of fit (GoF) test (*p* ≤ 0.05) indicated non-homogeneous variance, so we used univariate logistic regression. We reported the results of Fisher’s exact test as frequencies, percentages, and *p*-values, while the univariate logistic regression results were expressed as odds ratios (OR), *p*-values, and 95% confidence intervals (CI). To visualize the spatial distribution of cases, we produced a spatial map using ArcGIS version 10.2 (ESRI, 380 New York Street, Redlands, CA, 92373, USA).

## 3. Results

### 3.1. Spatial Distribution of Reported Sonali Cases

**Figure 1:**
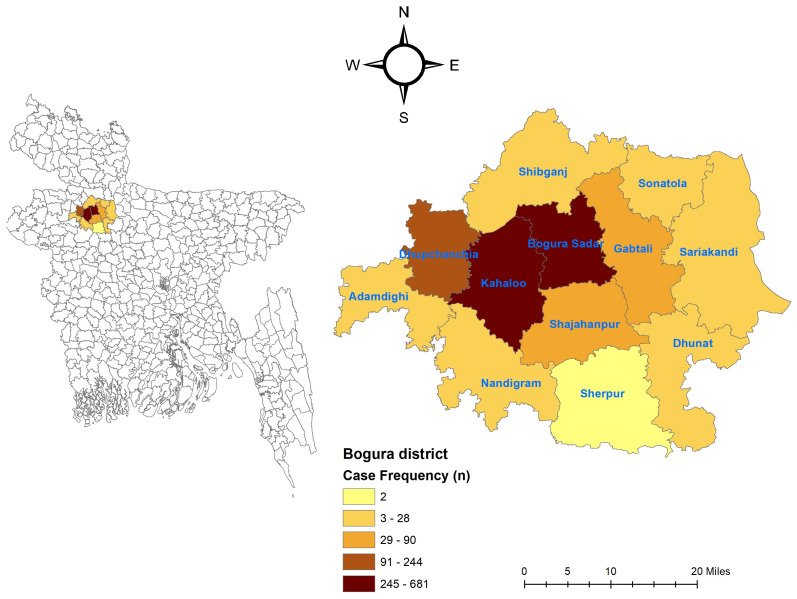
Spatial distribution. Distribution of 1690 Sonali chicken cases recorded on bdvets.com (2020–2021) across Bogura district, Bangladesh.

We found that Bogura Sadar upazila had the highest proportion of Sonali chicken disease cases, accounting for 40.3% of the total, followed by Kahaloo upazila with 28.2% and Dhupchanchia upazila with 14.4%. The remaining upazilas reported between 0.1% and 5.3% of cases. Specifically, we recorded 681 cases in Bogura Sadar, 477 in Kahaloo, and 244 in Dhupchanchia. Other upazilas reported the following case numbers: Sahajanpur (5.3%, 90 cases), Gabtali (4.1%, 69 cases), Shibganj (1.7%, 28 cases), Adamdighi (1.5%, 26 cases), Sariakandi (1.4%, 23 cases), Sonatola (1.1%, 18 cases), Dhunat (0.9%, 16 cases), Nandigram (0.9%, 16 cases), and Sherpur (0.1%, 2 cases) (Figure 1).

### 3.2. Distribution of Reported Cases by Different Associated Factors

**Figure 2:**
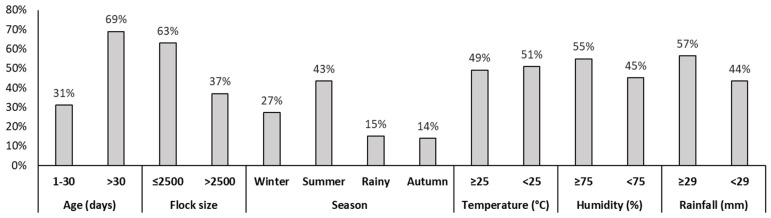
Distribution of 1690 Sonali cases recorded on bdvets.com (2020–2021) in Bogura district, Bangladesh, by age, flock size, season, temperature, humidity, and rainfall.

We observed a higher prevalence of infection in grower Sonali chickens than the starter phase (69% vs. 31%), and infection was more common in small to medium flocks than large flocks (63% vs. 37%). The highest number of cases occurred during the summer season (43%), followed by winter (27%), the rainy season (15%), and autumn (14%). Cases were more frequent at lower temperatures (<25 °C, 51%), high humidity (≥75%, 55%), and heavy rainfall (≥29 mm, 57%) (Figure 2).

### 3.3. Distribution of Sonali Cases Based on Diagnoses from Clinical Signs and Gross Necropsy Findings

ND was the most prevalent viral disease, accounting for 19.5% of cases. Other diseases identified included Marek’s disease (9.8%), coccidiosis (7.4%), necrotic enteritis (4.7%), infectious bursal disease (3.2%), and infectious laryngotracheitis (3.2%). Low pathogenic avian influenza and high pathogenic avian influenza were estimated at 2.3% and 0.1%, respectively (Table 1). Additionally, 31.4% of cases were classified as inconclusive (Table 1).

### 3.4. Univariate Analysis—ND Occurrence (Based on Clinical Signs and Gross Necropsy Findings) vs. Risk Factors

The occurrence of ND in Sonali chickens varied significantly by age, flock size, season, temperature, humidity, and rainfall (Table 2). These variables were, therefore, forwarded to construct the multivariate logistic regression.

### 3.5. Simple Logistic Regression Analysis—ND Occurrence (Based on Clinical Signs and Gross Necropsy Findings) vs. Risk Factors

The odds of ND were 1.4 times higher in grower chickens compared to starter chickens (*p* = 0.007). The odds were 11.4 times higher in summer, 4.1 times higher in autumn, and 3.9 times higher in the rainy season compared to winter (all *p* < 0.001). Chickens exposed to temperatures above 25 °C had 3.5 times higher odds of ND compared to those exposed to temperatures of 25 °C or below (*p* < 0.001). Chickens exposed to temperatures >25 °C had 3.5 times higher odds, and those exposed to humidity >75% had 2.6 times higher odds of ND (both *p* < 0.001) (Table 3).

### 3.6. Pattern of Antimicrobial Usage in Different Sonali Cases

Tylvalosin (38%) was the most prescribed antibiotic across different case types, followed by Fluoroquinolones (9%), Aminoglycosides (8%), Lincosamides (7%), Sulfa drugs (7%), Spectinomycin (6%), Colistin Sulphate (4%), Tylosin (4%), Tetracycline (3%), Florfenicol (2%), Doxycycline (1%), Penicillin (1%), Trimethoprim (1%), Fosfomycin (0.4%), and Cefuroxime (0.9%). Notably, 9% of the cases had no history of antibiotic use. Among other antimicrobials, Nicarbazin (5%) was frequently prescribed for coccidiosis and related cases, followed by Diclazuril (2%), Ivermectin (0.5%), and Amprolium (0.5%). Metronidazole, Nystatin, and Toltrazuril were prescribed in only 0.1% of cases (Table 4).

## 4. Discussion

The web-based passive surveillance platform bdvets.com serves as a private alternative to the government-led surveillance efforts, offering online prescriptions and support to remote farmers through both public and private veterinarians. While Bangladesh’s veterinary sector has made significant progress in improving surveillance and outbreak investigation, the DLS introduced the BAHIS platform, which currently does not collect data from non-governmental sources. In contrast, bdvets.com fills this gap by enabling 876 veterinarians (219 public and 657 private) to provide e-prescriptions after receiving reports of livestock and poultry diseases from farmers. Between November 2019 and 2021, a total of 1315 farmers (780 poultry farmers) used the platform’s services. Both veterinarians and farmers have access to the bdvets.com data recording system; however, farmers are limited to inputting demographic and case history information. Farmers can also choose a veterinarian for treatment or request an e-prescription via the platform, which helps improve disease management in remote areas through real-time data collection, early detection, and rapid response. Web-based passive surveillance systems like bdvets.com have become increasingly popular for their ability to enhance infectious disease management [36].

A total of 20 diseases and disease conditions that were presumptively diagnosed based on the clinical signs and gross necropsy findings were documented in Sonali chickens in the Bogura district of Bangladesh, occurring either individually or concurrently in this web-based passive surveillance. The most prevalent were viral diseases (notably ND and Marek’s disease), followed by bacterial infections (like necrotic enteritis, colibacillosis) and coccidiosis. These results align with the findings from several previous studies on Sonali and other poultry breeds/strains in Bangladesh [3,15,18,37,38]. Similar findings were reported in research conducted in developing countries like Nigeria, Pakistan, India, and Ethiopia [39,40]. However, some previous studies have found that Sonali chickens are more prone to coccidiosis than other viral and bacterial diseases, despite being affected by infectious diseases [3,15,16]. Differences in geographical locations, diagnostic procedures, and poor litter management are causes for the high prevalence of coccidiosis [15,19]. Several factors might have contributed to the wide range of viral diseases and/or disease conditions in this study, such as poor vaccination coverage, inappropriate vaccine usage, vaccine failure due to issues with the cold chain, subclinical conditions of the diseases, or inadequate sanitary and biosecurity practices [41,42]. In general, hygiene and biosecurity standards at Sonali chicken farms in Bangladesh are relatively poor [16].

The prevalence of ND at 19.5% underscores its critical threat to poultry health in Bangladesh. This disease is associated with high mortality and morbidity rates across various age groups, and its virulence has been noted to increase over time [22]. Several factors contribute to the severity of ND, including host-specific characteristics such as young age and immunosuppression as well as external conditions like environmental stress, temperature variations, and seasonal changes [43]. However, biosecurity practices are limited in small- and medium-scale poultry farms in Bangladesh, which can play a role in higher ND prevalence than the large-scale farms [44]. Continuous monitoring and systematic data collection from small-scale poultry farms are essential for understanding the dynamic epidemiology of ND, enabling the development of targeted control measures and mitigation strategies to lessen its impact on the poultry industry [45].

The observation of more ND cases in grower birds (31 days or more) in the present study is congruent with the findings of [46,47,48], who also reported more cases in grower and adult birds in Ethiopia and Nigeria. Grower birds might have longer exposure times than younger [49] and compromised space and management required [50], which could have increased the chance of more cases in grower birds.

In this study, ND was detected year-round, with significantly more cases in summer (OR = 11.4) compared to winter, aligning with the findings from [51], who reported a peak prevalence of 50.61% in Bangladesh and India during the summer. Similarly, Ref. [52] documented a high prevalence of ND in rural Libya, where the prevalence reached 55% in summer compared to 23% in winter. Another study in Bangladesh indicated summer prevalence rates of 6.37% compared to 4.98% during the rainy season and only 1.97% in winter [53]. In contrast, some research from Thailand reported the highest prevalence in the wet season [51], while a review found peaks during winter [51]. The increased ND prevalence during summer can be attributed to warmer temperatures and higher humidity, which create favorable conditions for the virus to thrive [53]. Additionally, heat stress can compromise the immune systems of birds, making them more susceptible to infections. Management practices, such as higher stocking densities during warmer months, further elevate the risk of disease transmission [53]. The prevalence of ND in commercial chickens, particularly specific breeds like Sonali, underscores the ongoing threat this disease poses to the poultry industry during the summer months [53]. The simple logistic regression model also indicated that more ND cases were found at ≥25 °C temperature (OR = 3.5) and ≥75% humidity (OR = 2.6) compared to <25 °C temperature and <75% humidity, respectively. No research on the effect of temperature and humidity on the ND prevalence in Sonali chickens was observed. But research on broiler chickens revealed similar findings in Iran [54], which suggested that reduction in feed intake and change in digestion due to temperature change affected ND prevalence. Antimicrobials are used to treat as well as prevent disease in the poultry sector in Bangladesh [55]. Antimicrobials, such as tetracycline, amoxicillin, tilmicosin, tylosin, tylvalosin, fluoroquinolones, colistin, and fosfomycin, are critically important for both human and animal health [24]. The widespread use of these medically significant antibiotics in commercial chicken farming contributes to the development and spread of antimicrobial resistance in microbial communities affecting both animals and humans [24]. The widespread use of these antibiotics is commonly used to address inadequate farm biosecurity and manage concurrent bacterial infections [55]. Moreover, due to a lack of proper diagnostic tools and testing, veterinarians often rely on clinical history, signs, and post-mortem lesions for diagnosing poultry diseases. Hence, achieving an accurate diagnosis can be difficult, which may result in irrational and inappropriate antibiotic prescriptions.

Finally, the bdvets.com system has proven to be a useful tool for recording disease and drug data. It can detect patterns in disease and antibiotic use within specific regions, which can be a valuable addition to the countries’ existing passive surveillance systems. However, there is no liaison between the government and this type of non-government initiative. We recommend building a strong reporting system and a good collaboration of data sharing between the Department of Livestock Services’ (DLS) existing data system, BAHIS, the bdvets.com, and the countries’ other public health related surveillance systems, which will ensure a better early disease emergence detection as well as keep a tab on other issues such as antimicrobial usage and resistance insurgence. It is also recommended to enhance the system of bdvets.com by integrating additional epidemiological data, such as farm and farmer demographics, biosecurity and management practices (e.g., vaccination), spatial and temporal information, and details on drug doses and durations.

This study focused exclusively on Sonali chicken cases within the Bogura district, as there were enough cases available for this research. One limitation of this study was the incomplete availability of epidemiological data (farm and farmer demographics, biosecurity and management practices (e.g., vaccination), spatial and temporal information, and details on drug doses and durations). The diagnosis of different Sonali chicken cases was primarily based on clinical signs and post-mortem lesions. While this approach may introduce certain limitations, such as potential information and recall biases, it remains a widely accepted and practical method in field settings where access to ancillary diagnostic tools is limited. Additionally, the inclusion of 31% of inconclusive cases could influence the observed case frequency. Despite these constraints, the diagnostic methodology provides valuable insights into the health status and disease patterns of Sonali chickens, forming a critical foundation for improving disease surveillance and control measures in Bangladesh. Moreover, only three veterinarians contributed to the 1690 case prescriptions in Bogura, which may have led to minor variations in prescription perceptions. However, it is worth noting that the data collection was conducted by registered and experienced veterinarians.

## 5. Conclusions

The findings of this study serve as a baseline for understanding the factors, especially the climate factors, affecting ND prevalence in Bangladesh based on clinical signs and gross necropsy findings, which will help in taking precautionary measures to control ND infections in poultry-dominated regions. The findings also highlight the prescribers’ perceptions regarding antibiotic use in different diseases and provide valuable insights for designing interventions and policies to promote responsible antimicrobial use in Bangladesh. Overall, this study underscores the potential of the web-based surveillance system to become part of the mainstream data collection system of the Department of Livestock Services (DLS). To enhance its effectiveness, the system should be improved to incorporate additional epidemiological data, contributing to the establishment of a real-time surveillance system. Furthermore, it is strongly recommended to establish and expand laboratory diagnostic facilities in field settings across Bangladesh, enabling more reliable and comprehensive disease surveillance and control efforts.

## Figures and Tables

**Table 1:** Distribution of 1690 Sonali cases recorded on bdvets.com (2020–2021) in Bogura district, Bangladesh (multiple counts were considered for cases with multiple diseases).

Categories	Name of the Disease	Frequency	%
Bacterial	Necrotic Enteritis	103	4.7
	Colibacilosis	23	1.1
	Salmonellosis	30	1.4
Viral	ND	426	19.5
	Mareks	214	9.8
	Infectious Bursal Disease	69	3.2
	Infectious Laryngotracheitis	69	3.2
	Low Pathogenic Avian Influenza	51	2.3
	High Pathogenic Avian Influenza	2	0.1
	Fowl pox	1	0.1
	Inclusion Body Hepatitis	1	0.1
	Lymphoid Leucosis	1	0.1
Protozoan	Coccidiosis	162	7.4
Miscellaneous	Mycoplasma	8	0.4
	Aspergillosis	1	0.1
	Gout	12	0.5
	Avitaminosis	11	0.5
	Chronic Respiratory Disease	8	0.4
	Worm infestation	1	0.1
Non-specific enteritis		309	14.1
Inconclusive cases		687	31.4

**Table 2:** Univariate association between ND and risk factors in 1690 Sonali chicken cases recorded on bdvets.com (2020–2021) across Bogura district, Bangladesh.

Variables	Categories	ND + Ve, n (%)	*p* (Fisher’s Exact Test)
Age (days)	1–30	153 (29.4)	0.009
	above 30	273 (23.3)	
Flock size	≤2500	270 (25.2)	1.000
	≥2500	156 (25.2)	
Season	Winter	201 (43.5)	<0.001
	Summer	154 (21)	
	Rainy	56 (21.9)	
	Autumn	15 (6.3)	
Temperature (°C)	≥25	305 (36.8)	<0.001
	<25	121 (14.1)	
Humidity (%)	≥75	307 (33.1)	<0.001
	<75	119 (15.6)	
Rainfall (mm)	≥29	335 (35.1)	<0.001
	<29	12.4)	

**Table 3:** Simple logistic regression model to identify the correlation between ND occurrence and associated risk factors in 1690 Sonali chicken cases recorded on bdvets.com (2020–2021) across Bogura district, Bangladesh.

Variables	Categories	OR	95% CI	*p* (LRT)
Age (days)	1–30	Ref		
	above 30	1.4	1.1, 1.7	0.007
Season	Winter	Ref		
	Summer	11.4	6.5, 21.4	<0.001
	Rainy	3.9	2.3, 7.4	<0.001
	Autumn	4.1	2.2, 8.2	<0.001
Temperature (°C)	<25	Ref		
	≥25	3.5	2.7, 4.5	<0.001
Humidity (%)	<75	Ref		
	≥75	2.6	2.1, 3.4	<0.001

**Table 4:** Distribution of antimicrobials prescribed in 1690 Sonali chicken cases recorded on bdvets.com (2020–2021) in Bogura district, Bangladesh (multiple counts were considered for cases where antimicrobials were used more than once).

Name of the Antimicrobials (Name/Group/Class)	Frequency	%
Aminoglycosides #	158	8.3
Cefuroxime	1	0.1
Colistin sulphate *	74	3.9
Doxycycline	25	1.3
Florfenicol	36	1.9
Fluoroquinolones ¶*	167	8.9
Fosfomycin *	7	0.4
Lincosomide	129	6.9
Penicillin	24	1.3
Spectinomycin	104	5.5
Sulfa drugs	133	7.1
Tetracycline	48	2.6
Trimethoprim	23	1.2
Tylosin *	70	3.7
Tylvalosin §*	722	38.4
Amprolium	9	0.5
Diclazuril	37	2.0
Ivermectin	10	0.5
Metranidazole	1	0.1
Nicarbazin	101	5.4
Nystatin	1	0.1
Toltazuril	1	0.1
No antimicrobial	168	8.9

[* = Critically important antimicrobials classified as per WHO Critically Important Antimicrobials for Human Medicine 6th revision; # = Common Aminoglycosides were gentamicin, neomycin, and streptomycin; ¶ = Common fluoroquinolones were ciprofloxacin, levofloxacin, ofloxacin, nalidixic acid, pefloxacin, and sparofloxacin; § = Tylvalosin derived from tylosin through the modification of 3-acetyl-4′-isovaleryl (acetylisovaleryltylosin tartrate)].

## Data Availability

Some of the original data presented in this study are not publicly available and were collected from bdvets.com. Further information regarding the data can be requested from the corresponding author.
